# A genomic data viewer for iPad

**DOI:** 10.1186/s13059-015-0595-3

**Published:** 2015-02-26

**Authors:** Helga Thorvaldsdóttir, James T Robinson, Douglass Turner, Jill P Mesirov

**Affiliations:** Broad Institute of MIT and Harvard, Cambridge, MA 02142 USA

**Keywords:** Genomic data viewer, Visualization, Next-generation sequencing, Mobile, iPad, Open source software

## Abstract

The *Integrative Genomics Viewer (IGV) for iPad*, based on the popular IGV application for desktop and laptop computers, supports researchers who wish to take advantage of the mobility of today’s tablet computers to view genomic data and present findings to colleagues.

The computing landscape has changed significantly in the last few years. Mobile devices are now ubiquitous, extending the online experience beyond desktop computers. While mobile devices are not yet capable of performing the compute-intensive analyses required for most genomic research, they are capable of supporting data visualization and the presentation of findings. For this purpose, we developed the *Integrative Genomics Viewer (IGV) for iPad*, an open-source lightweight viewer for browsing genomic data on Apple iPad tablets. IGV for iPad is based on our popular IGV application for desktop and laptop computers [1,2] that is known for efficient visualization of large heterogeneous datasets, allowing the user to zoom and pan seamlessly across the genome at all levels of resolution. IGV for iPad offers a select subset of commonly used data types and features from desktop IGV, keeping a similar visual appearance of the data layout and representation, while making full use of the iOS user interface features that iPad users expect.

## Loading data

**Figure 1:**
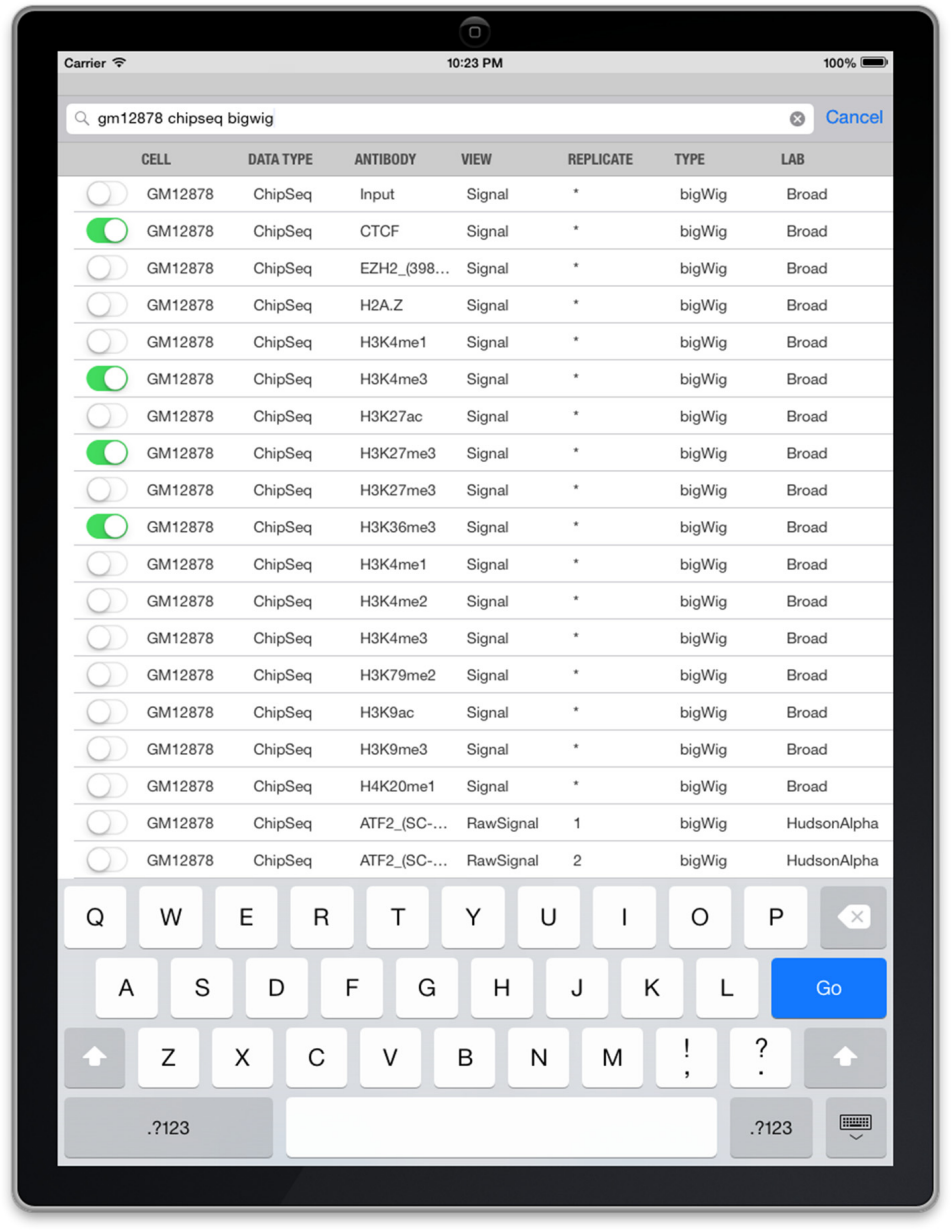
**Searchable menu for ENCODE tracks.** A table lists the datasets available on the ENCODE project website, showing only those that can be viewed in IGV for iPad. The user can also search and filter on dataset attributes to narrow down the dataset choices before selecting one or more to load.

IGV for iPad supports a variety of data types that can be displayed in the context of genomic coordinates, for example, DNA and RNA sequence alignments, copy number, mRNA abundance, ChIP-Seq data, and others. The *Tracks > Public Tracks* menu lists a number of sample public datasets that are relevant for the currently selected reference genome. These include DNA sequencing data from the 1000 Genomes project [3], RNA sequencing data from the Human lincRNA Catalog [4,5], based on Illumina’s Human BodyMap 2.0 data [6], and production data from The Encyclopedia of DNA Elements (ENCODE) Consortium [7] project website [8]. As the ENCODE data include more than 20,000 files, IGV for iPad automatically filters the ENCODE track menu (see Figure 1) to show only data types supported by the app, including raw signals, processed peaks, splice junctions sites, sequencing alignments, and more. Entering one or more terms into a search box will further filter the datasets, and at any point, the user can select one or more of the datasets to load into IGV for iPad.

IGV for iPad is not limited to the datasets provided in the *Public Tracks* menu. A user can load any dataset through the *Tracks > My Tracks* menu, as long as the file is accessible via a web address (URL) and is in one of the supported file formats, which currently include BAM [9], WIG [10], BIGWIG [11], TDF [12], SEG [13], and BED [14]. The file can be hosted on the web or on a local intranet, and the mechanism for making the file accessible is independent of IGV. However, as a convenience for our users, on our website we provide step-by-step instructions for some easy-to-use options for sharing data on the web (see [15]). These options include: (i) Dropbox [16], a popular commercial web-based data-hosting service; (ii) Simple Storage Service (S3), a data-hosting service provided by Amazon Web Services [17]; and (iii) GenomeSpace [18], a freely-available environment for integrative genomics analysis, which also allows its users to upload data files to cloud storage and share them with collaborators or make them publicly available.

## Viewing data

Once the data are loaded into the app, familiar touch-based gestures are used to interact with the view. For example, swiping will pan across the genome, and tapping on the chromosome ideogram will center the view on the selected region. Pinching in and out changes the zoom level, but a slider also provides the convenience of moving quickly through many levels of genome resolution. Entering a locus or gene name in a search box will zoom to the specified region.

**Figure 2:**
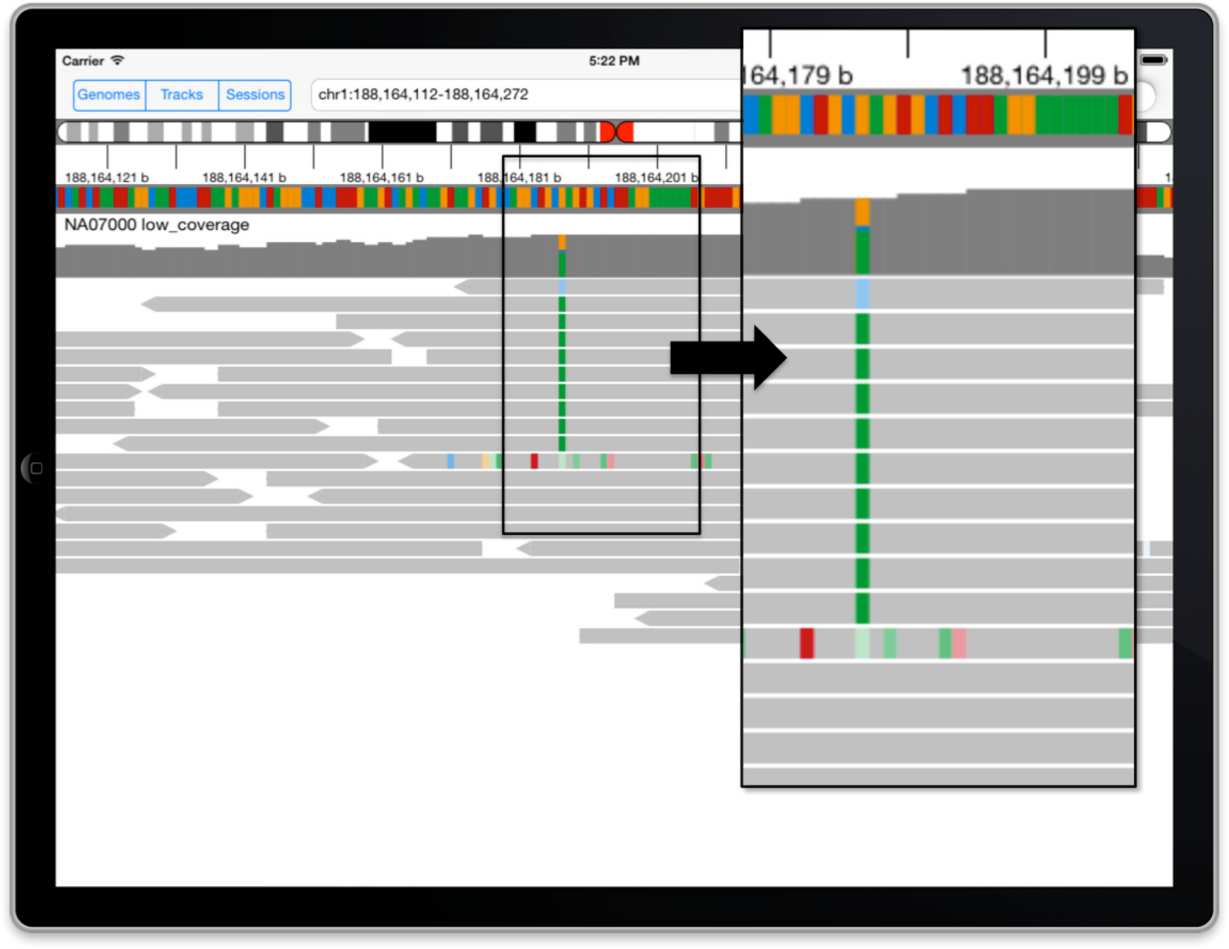
**DNA sequencing data.** Color is used to highlight variant bases in the grey bars representing aligned reads. Sorting the reads by base can further highlight a putative SNP.

**Figure 3:**
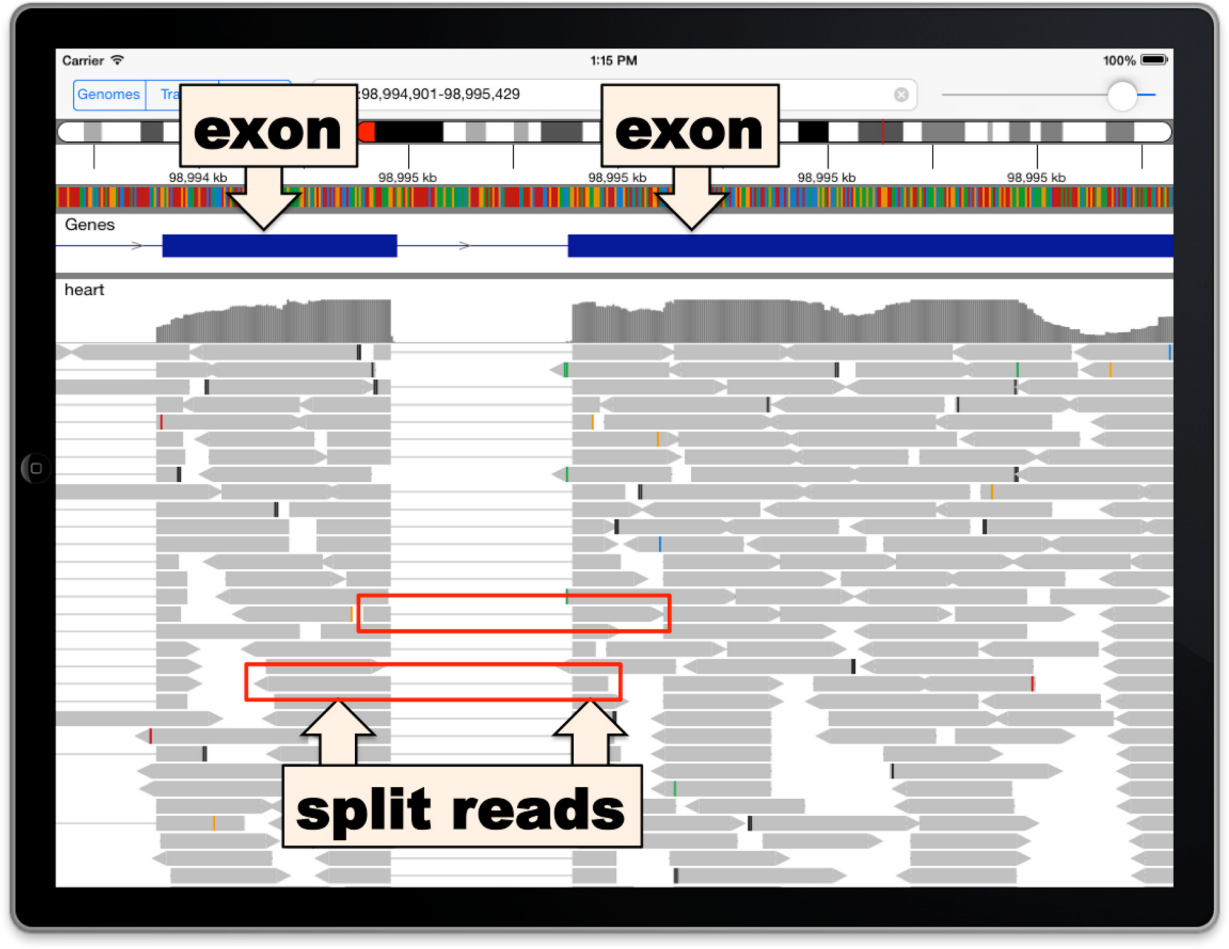
**RNA sequencing data.** Thin lines connect RNA read segments across splice junctions.

Data tracks in IGV for iPad are very similar in appearance to IGV on desktop computers. Aligned sequencing reads are drawn as grey horizontal bars, with variant bases highlighted in color. Color intensity indicates the quality of the base call. Sorting the reads by base is a useful tool when viewing the sequencing data supporting a putative single-nucleotide polymorphism (SNP) (see Figure 2). When RNA sequencing reads span exon junctions, they are split into segments when aligned to the reference genome sequence. IGV connects these split read segments with a thin line across introns (see Figure 3). IGV dynamically computes the depth of read coverage in the viewed region and displays a coverage bar chart in addition to the individual reads. The coverage chart also highlights with color any loci where a significant number of reads do not match the reference genome. Segmented copy number data are drawn as horizontal bars, with a red/blue heatmap coloring scheme where the hue and intensity indicate the copy number level. Numeric data are presented as vertical bars, with the height representing the data value.

## Launching IGV for iPad from web links

**Figure 4:**
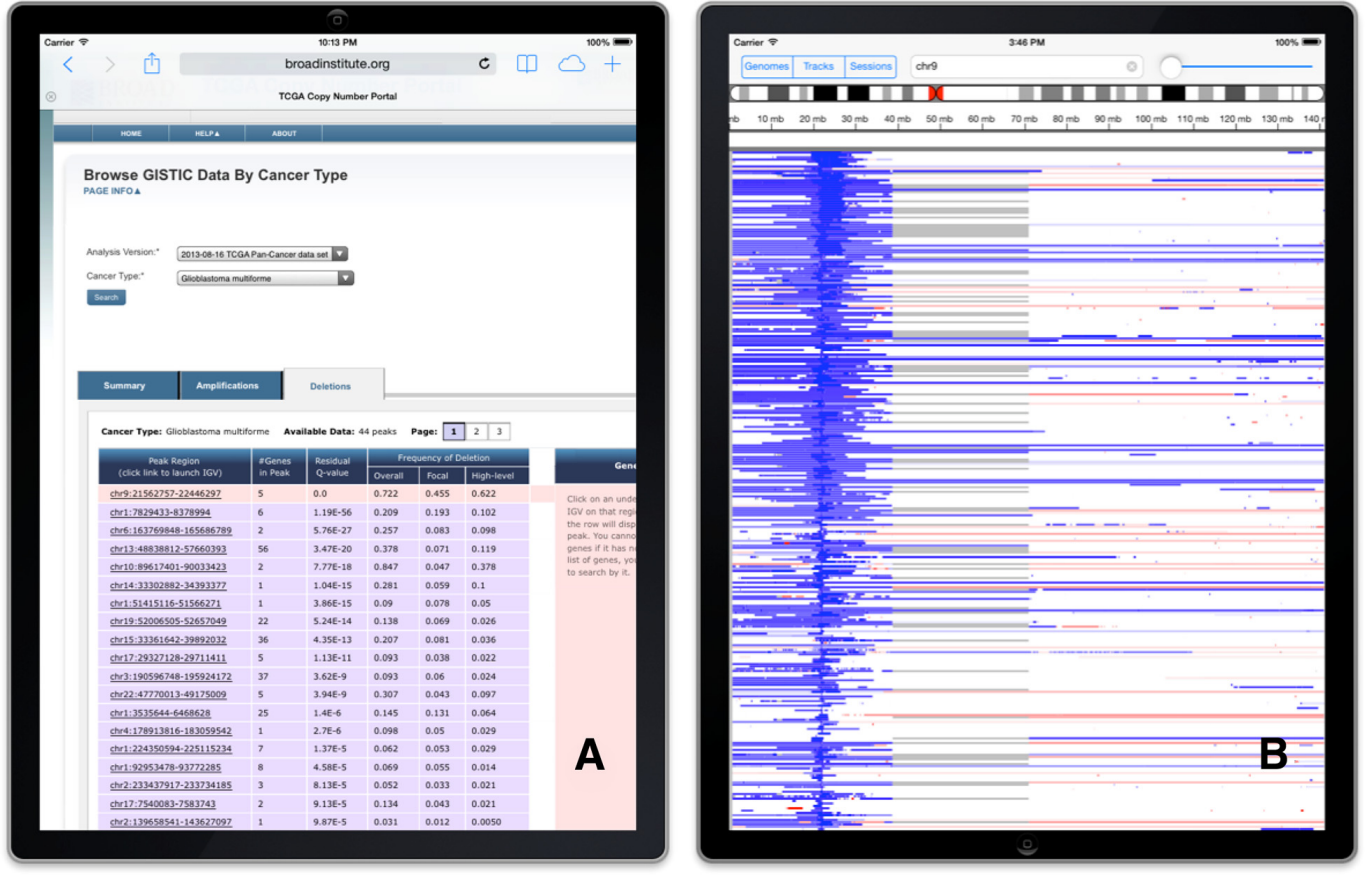
**Segmented copy number data. A**. IGV can be launched directly from the TCGA Copy Number Portal. **B**. Copy number data, with samples sorted by deletion.

In addition to starting the app from the icon on the iPad Home screen, users can launch IGV for iPad from links embedded in web pages, documents, and email messages. The same links will launch IGV on an iPad or a desktop computer, depending on the user’s device. The links can also specify the reference genome to use, one or more datasets, and the initial viewing locus. Importantly, users can easily create these links to serve as “bookmarks” to views of their datasets and then share them privately with colleagues or publicly with the scientific community. A number of genomic data web portals take advantage of these links to allow their users to launch IGV to view specific events of interest in the data. For example, The Cancer Genome Atlas (TCGA) [19] Copy Number Portal [20] presents somatic copy number alterations across multiple cancer types from data generated at the Broad Institute TCGA Genome Characterization Center. The portal allows users to query pre-defined analyses to see copy number alterations affecting an individual gene across multiple cancer types, and significant regions of amplification and deletion in individual cancer types. When viewing the portal website on an iOS device, tapping on links in the query results will launch IGV for iPad to display the underlying segmented copy number data (see Figure 4). A touch-and-hold gesture on any data track in the app brings up a context sensitive menu. For segmented copy number data, the menu includes an option to sort the samples by the level of amplification and deletion. In the figure, the samples have been sorted by deletion.

## Challenges

Development of a genomic viewer for a mobile platform presents challenges due to limited memory and disk space in comparison with desktop and laptop computers. Also, the iPad device does not have a general-purpose file system for storing and managing data files. Data are loaded into IGV for iPad across the wireless network. To minimize data transfer and memory usage, we drew on lessons learned from our experience developing desktop IGV:

1. We implemented support for file formats that were designed to allow reading relatively small portions of the file at a time, so the app can load data only as needed when regions come in to view. Examples of data types that can take advantage of these formats include large gene annotation files such as the RefSeq gene track (indexed BED format), aligned reads from next-generation sequencing (BAM format), ChIP-Seq signal data (TDF and BIGWIG formats), and whole-genome sequencing coverage (TDF and BIGWIG formats). We note that IGV for iPad also supports the simpler plain WIG and BED formats for smaller datasets.
2. For aligned sequence data we only retrieve data from the source file when the view has been zoomed in to a sufficiently small region so that individual aligned reads (on the order of tens of bases in length) can be distinguished. Also, the app reduces the number of reads kept in memory by using a reservoir sampling method to randomly choose alignments to draw, and discards all others. The bar chart showing the read coverage at each locus is computed before any reads are discarded and always shows the true coverage levels. Parameter settings for both the zoom visibility threshold and the down sampling can be adjusted in the IGV section of the iPad System Preferences.

## Concluding remarks

We have found very few genomic data viewers for the iPad, and none that are open source. The GeneWall app from Wobblebase Inc. is designed for exploring public gene annotations. Their premium for-fee version also allows uploading personal genome information from companies such as 23andMe and Complete Genomics, as well as additional annotation tracks in simple BED format. The Genome Wowser app, from the Center for Biomedical Informatics at The Children’s Hospital of Philadelphia Research Institute, was available in the Apple App Store until recently. It provided an iPad interface to a number of tracks from the University of California Santa Cruz (UCSC) Genome Browser, but it did not support viewing data from any other sources.

To our knowledge, IGV is the only iPad app that supports loading and viewing a variety of types of user-generated genomic data. IGV for iPad can load reference genomes and datasets from any web-accessible source, thereby allowing researchers to visualize and present findings in their own data. Taking advantage of the flexibility of web links to launch the app with pre-loaded data at a specified locus, investigators can easily share their findings by adding these links to their websites and research documents.

IGV for iPad was developed using the iOS native language, Objective C, and the app is freely available for download from the Apple App Store. The software is released under a Massachusetts Institute of Technology (MIT) open source license, and hosted on GitHub at [21]. User documentation can be found on the IGV website at [22].

## Abbreviations

ENCODE: Encyclopedia of DNA elements
IGV: Integrative genomics viewer
SNP: Single nucleotide polymorphism
TCGA: The Cancer Genome Atlas
